# Drug Transporters in the Kidney: Perspectives on Species Differences, Disease Status, and Molecular Docking

**DOI:** 10.3389/fphar.2021.746208

**Published:** 2021-11-29

**Authors:** Wei Zou, Birui Shi, Ting Zeng, Yan Zhang, Baolin Huang, Bo Ouyang, Zheng Cai, Menghua Liu

**Affiliations:** ^1^ Changsha Research and Development Center on Obstetric and Gynecologic Traditional Chinese Medicine Preparation, NHC Key Laboratory of Birth Defects Research, Prevention and Treatment, Hunan Provincial Maternal and Child Health Care Hospital, Changsha, China; ^2^ Biopharmaceutics, NMPA Key Laboratory for Research and Evaluation of Drug Metabolism, School of Pharmaceutical Sciences, Southern Medical University, Guangzhou, China; ^3^ TCM-Integrated Hospital, Southern Medical University, Guangzhou, China

**Keywords:** renal drug transporters, species, sex-genders, ages, molecular docking, disease status

## Abstract

The kidneys are a pair of important organs that excretes endogenous waste and exogenous biological agents from the body. Numerous transporters are involved in the excretion process. The levels of these transporters could affect the pharmacokinetics of many drugs, such as organic anion drugs, organic cationic drugs, and peptide drugs. Eleven drug transporters in the kidney (OAT1, OAT3, OATP4C1, OCT2, MDR1, BCRP, MATE1, MATE2-K, OAT4, MRP2, and MRP4) have become necessary research items in the development of innovative drugs. However, the levels of these transporters vary between different species, sex-genders, ages, and disease statuses, which may lead to different pharmacokinetics of drugs. Here, we review the differences of the important transports in the mentioned conditions, in order to help clinicians to improve clinical prescriptions for patients. To predict drug-drug interactions (DDIs) caused by renal drug transporters, the molecular docking method is used for rapid screening of substrates or inhibitors of the drug transporters. Here, we review a large number of natural products that represent potential substrates and/or inhibitors of transporters by the molecular docking method.

## Introduction

**FIGURE 1:**
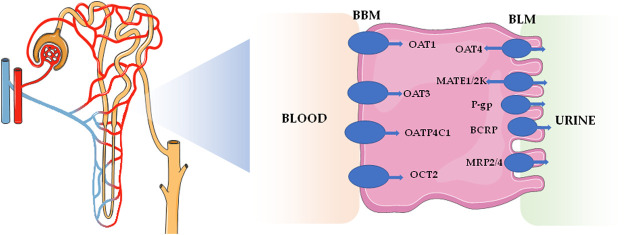
Distribution of the major transporters located in kidney.

The kidneys are the main excretory organs of the body, which play key roles in excretion of metabolites, acid-base balance, and homeostasis of the body system. The secretion and reabsorption effects of renal tubules are mainly mediated by transporters. It is not only an effective mechanism for the reabsorption of nutrients, such as glucose and amino acids, but also an effective way to remove endogenous waste and exogenous biological agents. Up to now, renal excretions of many drugs (including organic anion drugs, organic cationic drugs, and peptide drugs) are mediated by drug transporters concentrated on proximal renal tubules (Ivanyuk et al., 2017). On the one hand, uptake transporters, such as organic anion transporters (OATs) and organic cationic transporters (OCTs), on the basolateral membrane of renal tubular epithelial cells take up drugs from the blood side into cells. On the other hand, efflux transporters, such as multidrug and toxin extrusion proteins (MATEs) and multidrug resistance proteins (MRPs), located on the brush edge of renal tubular epithelial cells discharge intracellular drugs into the lumen for secretion and excretion (Gozalpour and Fenner, 2018). The study on drug transporters has become one of the main trends in the field of pharmacokinetics. Impacts on 11 drug transporters in the kidneys, including OAT1, OAT3, organic anion transporter polypeptide 4C1 (OATP4C1), organic cation transporter (OCT2), multidrug resistance protein 1 [MDR1, namely p-glycoproteins (P-gp)], breast cancer resistance protein (BCRP), MATE1, MATE2-K, OAT4, multidrug resistance-associated protein 2 (MRP2), and MRP4, have become necessary research items in the development of innovative drugs (Food and Drug Administration, 2020). These drug transporters are mainly distributed on the basolateral membrane and apical membrane of renal proximal tubular cells, which are shown in Table 1 and Figure 1.

**TABLE 1:** The main drug transporters on the proximal tubular cells.

Protein	Full name	Location	Gene in human	UniProt ID
OAT1	Organic anion transporter 1	Basolateral membrane	*SLC22A6*	Q4U2R8
OAT3	Organic anion transporter 3	Basolateral membrane	*SLC22A8*	Q8TCC7
OATP4C1	Organic anion transporter polypeptide 4C1	Basolateral membrane	*SLCO4C1*	Q6ZQN7
OCT2	Organic cation transporter	Basolateral membrane	*SLC22A2*	O15244
MDR1	Multidrug resistance protein 1	Apical membrane	*ABCB1*	P08183
BCRP	Breast cancer resistance protein	Apical membrane	*ABCG2*	Q9UNQ0
MATE1	Multidrug and toxin extrusion protein 1	Apical membrane	*SLC47A1*	Q96FL8
MATE2-K	Multidrug and toxin extrusion protein 2-k	Apical membrane	*SLC47A2*	Q86VL8
OAT4	Organic anion transporter 4	Apical membrane	*SLC22A11*	Q9NSA0
MRP2	Multidrug resistance protein 2	Apical membrane	*ABCC2*	Q92887
MRP4	Multidrug resistance protein 4	Apical membrane	*ABCC4*	O15439

UniProt: https://www.uniprot.org/

At present, pharmacokinetic data in drug instructions are primarily derived from healthy subjects, and most pharmacokinetic experiments are performed in healthy adult animals to evaluate drug safety and *in vivo* processes (absorption, distribution, metabolism, and excretion). However, two issues require attentions: 1) Which characteristics of the animal model (such as species, age, sex-gender, and disease status) can best reflect pharmacokinetic behaviour in humans? 2) Does the pharmacokinetic data from healthy subjects (human and/or animal) appropriately reflect the disease status? Extensive literature in recent decades has shown that the changes of drug transports in the disease status will lead to the changes of the drug pharmacokinetic behaviour, which directly affect the performance of drug efficacy or produce toxicities and side effects (Yang and Han, 2019). Therefore, it is very important to understand the changes of transporters in the disease status for optimizing clinical drug administration. In addition, patients often take multiple drugs simultaneously in clinic, and then drugs may cause drug-drug interactions (DDIs), which result in serious adverse reactions or an altered treatment outcome. A statistical study of hospitalized elderly patients has revealed that the prevalence of DDIs ranged from 8.34 to 100% (de Oliveira et al., 2021). Drug transporter variation is one of the mechanisms of DDIs, as the drugs may be substrates or mediators of the drug transporters. At present, computer-based drug design is widely used in drug development to simulate the chemical structural interactions between biomacromolecules and drugs, and hence, this technique has been used in transporter studies to quickly predict DDIs in clinic (Marques et al., 2019).

In this review, we mainly discuss the renal transporter expression differences on species, sex-gender, and age, as well as the changes of drug transporters under disease statuses. Furthermore, the molecular docking technology applied on renal drug transporters was reviewed, which could facilitate the prediction of DDIs for improving the safety and effectiveness of drugs in clinic.

## The Expressions of Drug Transporters in Kidney Across Species, Sex-Gender, and Age

### Species

Preclinical trials are essential for evaluating drug safety, efficacy, toxicity, and pharmacokinetics. It is necessary to understand the difference between humans and animals at the levels of drug transporters. Along with quantitative real time-polymerase chain reaction (qPCR) and Western blot, the highly sensitive liquid chromatography tandem-mass spectrometry (LC-MS/MS) has become one of the most effective methods in the quantitative detection of drug transporters (Fallon et al., 2016; Nakamura et al., 2016; Basit et al., 2019). Table 2 has shown the species differences of renal drug transporters found in past decades. Basit et al. (2019) showed that the abundances of OAT1, OCT2, and MATE1 in human renal tissue were higher than the other eight drug transporters. The levels of OAT1, OAT3, OATP4C1, OCT2, MDR1, MATE1, OAT4, MRP2, and MRP4 in monkey were from 1.6-fold to 3.7-fold higher than that in human. OATP4C1, MATE1, MATE2-K, OAT4C1, and MRP2 in rat and mouse, as well as OATP4C1, OCT2, MATE1, MATE2-K, OAT4C1, MRP2, and MRP4 in dog, were not detected as no conserved peptide available (Basit et al., 2019). Fallon et al. (2016) have revealed three renal efflux transporters in human, monkey, dog, and rat. The concentrations of MDR1 and MRP2 were similar in human and monkey, but higher than that in rat. However, the BCRP level was the highest in rat (4.5 pmol mg^−1^ protein), which is 50-fold than that in human (0.09 pmol mg^−1^ protein). In another study, 11 transporters were detected in the pooled microsomal fraction of human kidney (Nakamura et al., 2016). All transporters, except for OATP4C1, have sensitive responses when detection. The content of BCRP (3.6–4.5 pmol mg^−1^) was similar as the other reports (Fallon et al., 2016; Nakamura et al., 2016; Al-Majdoub et al., 2020). Many exogenous substances show species differences in excretion. Taking per- and polyfluoroalkyl substances (PFAS) as an example, a longer serum half-life in human (t_1/2_), compared to that in animal species, is potentially due to the difference in excretion which was mediated by renal tubular OATs and OATPs (Ducatman et al., 2021). Therefore, the differences on cross-species kidney transporters should be carefully considered in drug preclinical-to-clinical stage.

**TABLE 2:** The expressions of main drug transporters in different species and sex-genders.

Species	Method	Units	Transporters											Reference
			OAT1	OAT3	OATP4C1	OCT2	MDR1	BCRP	MATE1	MATE2-K	OAT4	MRP2	MRP4	
Human	LC-MS/MS	pmol g^−1^	107.7 ± 56.83	78.5 ± 37.38	0.3 ± 0.03	164.2 ± 53.27	42.3 ± 16.16	BLQ	105.6 ± 47.52	—	10.6 ± 5.64	30.1 ± 16.52	19.5 ± 20.58	Basit et al. (2019)
	LC-MS/MS	pmol mg^−1^	—	—	—	—	3.89 ± 1.30	—	—	—	—	0.10 ± 0.08	0.15 ± 0.08	Fallon et al. (2016)
	LC-MS/MS	fmol μg^−1^ of microsomes	5.31 ± 0.07	9.68 ± 0.18	BLQ	5.12 ± 0.07	4.45 ± 0.07	0.66 ± 0.02	10.8 ± 0.1	2.19 ± 0.05	1.56 ± 0.03	1.04 ± 0.02	1.49 ± 0.04	Nakamura et al. (2016)
	LC-MS/MS	pmol mg^−1^	—	—	—	—	3.63 ± 1.14	0.09 ± 0.02	—	—	—	0.48 ± 0.27	—	Al-Majdoub et al. (2020)
sex	LC-MS/MS	—	↔	↔	ND	↔	↔	BLQ	↔	—	↔	↔	↔	Basit et al. (2019)
Monkey	LC-MS/MS	pmol g^−1^	242.5 ± 62.69	124.7 ± 32.57	0.7 ± 0.28	464.8 ± 147.18	52 ± 9.44	BLQ	161.2 ± 56.23	—	17.5 ± 6.28	56 ± 13.99	71.3 ± 18.73	Basit et al. (2019)
	LC-MS/MS	pmol mg^−1^	—	—	—	—	3.05 ± 2.11	0.42 ± 0.36	—	—	—	0.52 ± 0.46	—	Fallon et al. (2016)
sex	LC-MS/MS		↔	↔	↔	↔	↔	BLQ	↔	—	↔	↔	↔	Basit et al. (2019)
Dog	LC-MS/MS	pmol g^−1^	75.4 ± 43.07	NC	NC	NC	32.1 ± 9.34	NC	NC	—	NC	NC	NC	Basit et al. (2019)
	LC-MS/MS	pmol mg^−1^	—	—	—	—	1.07	0.15	—	—	—	0.55	—	Fallon et al. (2016)
sex	LC-MS/MS	—	↔	NC	NC	NC	F>M*1.4	NC	NC	—	NC	NC	NC	Basit et al. (2019)
Rat	LC-MS/MS	pmol g^−1^	308.8 ± 79.24	NC	NC	253.5 ± 70.92	39.3 ± 11.76	1.3 ± 0.4	NC	—	NC	NC	37.5 ± 7.51	Basit et al. (2019)
	LC-MS/MS	pmol mg^−1^	—	—	—	—	1.74 ± 1.09	4.50 ± 2.88	—	—	—	0.27 ± 0.21	—	Fallon et al. (2016)
	LC-MS/MS	fmol mg^−1^	10.5 ± 1.1	6.71 ± 1.03	—	—	0.682 ± 0.103	15.9 ± 1.5	2.04 ± 0.18	—	—	—	0.539 ± 0.090	—
sex	—	—	M > F*1.3	NC	NC	M > F*1.4	M > F*1.6	M > F*1.6	NC	—	NC	NC	↔	Basit et al. (2019)
Mouse	LC-MS/MS	pmol g^−1^	156.2 ± 92.06	NC	NC	429.1 ± 134.67	15.5 ± 5.99	3.1 ± 0.88	NC	—	NC	NC	8.6 ± 3.94	Basit et al. (2019)
sex	LC-MS/MS	—	M > F*3.2	NC	NC	M > F*1.6	F > M*2.0	↔	NC	—	NC	NC	F > M*2.4	Basit et al. (2019)
	qPCR	RLU/10 μg	M > F	↔ in C57BL/6 mice	—	—	—	—	—	—	—	—	—	Buist and Klaassen, (2004)
	—	—	—	M < F in 129 J mice	—	—	—	—	—	—	—	—	—	—

NC: no conserved peptide; ↔: No significant difference; ND: sex difference was not determined; BLQ: below limit of quantification; /: not mentioned.

### Sex-Gender and Pregnancy

As an important factor, sex-gender should attract a full attention in the context of drug development and clinical use. Between 1995 and 2000, 11 out of 300 new drug applications recorded by the Food and Drug Administration (FDA) showed >40% sex-gender differences in pharmacokinetics (Anderson, 2005). Taking methotrexate as an example, after 1,277 osteosarcoma patients were treated with a high dose of methotrexate, the incidence of delayed excretion in female patients was about 1.75%, which was significantly higher than that in male patients (0.37%) (Zhang et al., 2016). It has been reported that OAT1, OAT3, OATP4C1, OCT2, MDR1, MATE1, OAT4, MRP2, and MRP4 in the kidney show no sex-gender differences in healthy monkeys. However, significant sex-gender differences of OAT1, OCT2, MDR1, BCRP, and MRP4 are observed in rats and/or mice with different trends (Buist and Klaassen, 2004; Basit et al., 2019). For example, the abundance of MDR1 was 1.6-fold greater in male rats than that in the females, whereas its expression was twice as high in female mice compared with that in the males. It has been shown that OATP4C1, BCRP, and MATE2-K levels exhibited sex-based differences between healthy men and women (Basit et al., 2019). Due to sex-gender differences in transporter expression, full consideration should be given to pharmacokinetic, safety, efficacy, and toxicity studies of drug to allow better interpretation of clinical data. For example, 2 h after oral administration of metformin at a dose of 500 mg/kg, the cumulative urinary excretion and renal tissue-to-plasma concentration ratio in female rats (26,689 ± 1,266 µg and 2.96 ± 0.47 ml/g, respectively) were markedly lower than that in male rats (32,949 ± 1,384 µg and 4.20 ± 0.31 ml/g, respectively), which are potentially explained by gender-related differences on renal OCT2 expression, as the metformin is the substrate of OCT2 (Ma et al., 2016). Pregnancy is an especial physiological status for women. Lu et al. have proved no significant differences among OAT1/3, OCT2, MRP2, and MATE1 mRNA expressions in the kidneys between pregnant and non-pregnant rats (Lu et al., 2020).

### Age

**FIGURE 2:**
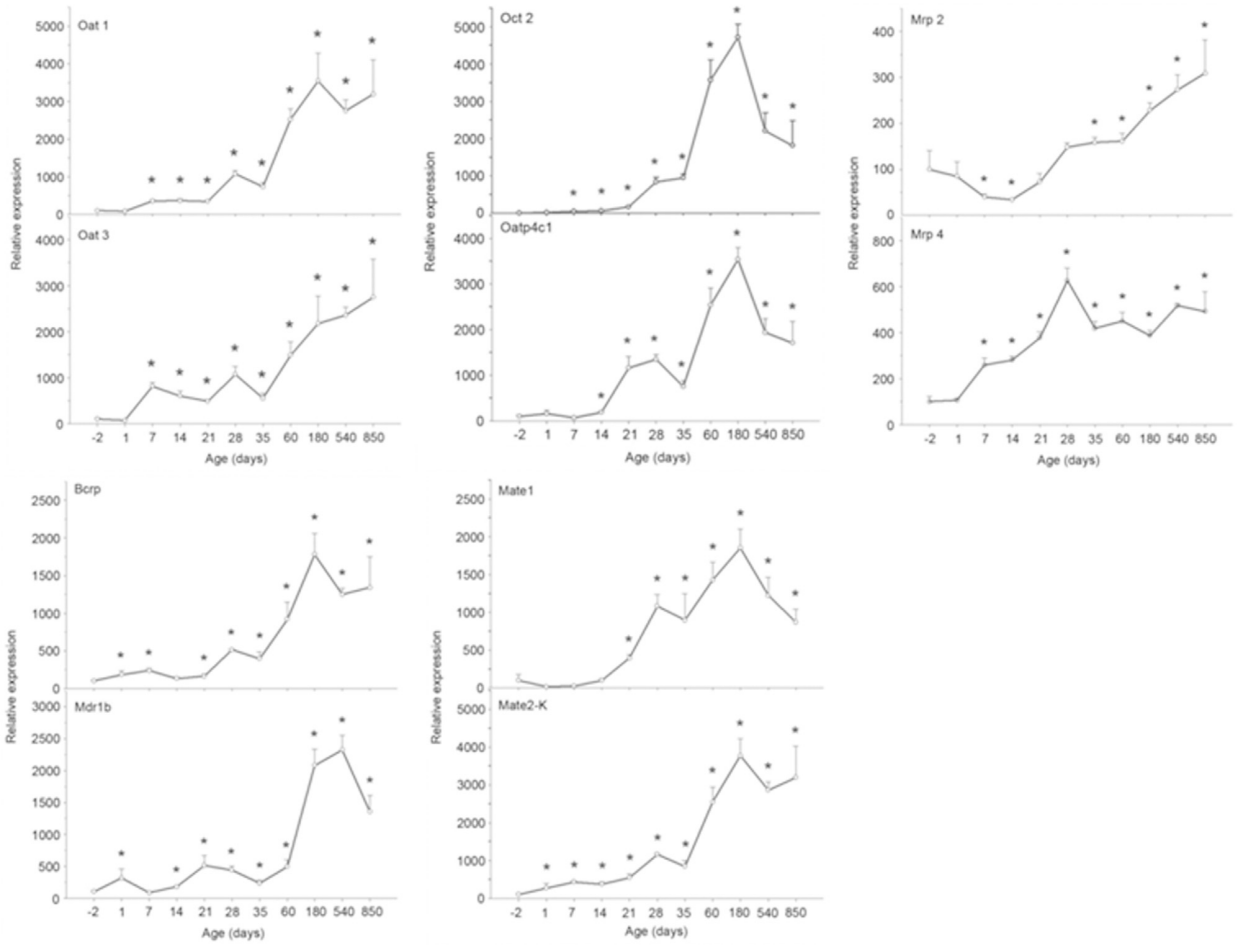
The mRNA expressions of OAT1, OAT3, OATP4C1, OCT2, MDR1, BCRP, MATE1, MATE2-K, MRP2, and MRP4 in rat kidney at the −2nd, 1st, 7th, 14th, 21st, 28th, 35th, 60th, 180th, 540th, and 850th day. Data are expressed as the mean ± standard error (*n* = 6). *p < 0.05 vs. the −2nd day (Anderson, 2005).

Age affects the transcription and translation of drug transporters. Joseph et al. (2015) have determined 30 drug transporters in two groups of human kidneys (age <50 years and ≥50 years). Among them, the mRNA expression of the OCT2 in the <50 years group was greater than that in the ≥50 years group (Joseph et al., 2015). The nephrotoxicity of cisplatin exhibits an age dependence in human. Wen et al. (2015) had analyzed 182 cisplatin-treated patients to illuminate the influence of nongenetic factors on cisplatin-induced nephrotoxicity, and age (≥50 years) is closely associated with the decline of renal function with an odds ratio (OR) of 11.771. This might be due to the fact that the MATE1, which excretes cisplatin into urine, was significantly decreased with the increasing age (≥50 years), resulting in the specific accumulation of cisplatin in renal cells. Although it is difficult to investigate the changes of drug transporters in the human kidney throughout life, an age-related study on OAT1, OAT3, OATP4C1, OCT2, MDR1, BCRP, MATE1, MATE2-K, MRP2, and MRP4 mRNA expressions was performed in male rat kidneys with qPCR and Western blot methods (Xu et al., 2017). In this preclinical study, all the detected drug transporters showed different increasing trends. As shown in Figure 2, the mRNA expressions of OAT1, OATP4C1, OCT2, BCRP, MATE1*,* and MATE2-K reached the peaks at the 180th day and subsequently declined. Compared with the mRNA expressions at the -2nd day and/or the 1st day, the expressions at the peak were 35-fold, 35-fold, 470-fold, 18-fold, 18-fold, and 37-fold greater for six drug transporters, respectively. The mRNA expressions of OAT3 and MRP2 reached the peak at the 850th day. For OAT3, the mRNA expression at the 180th day was 22-fold greater than that at the 1st day, while it was 3-fold greater at the 850th day than that at the -2nd day for MRP2. Interestingly, the mRNA expression of MRP2 was reduced almost 50% at the 14th and the 21st day compared with the -2nd day. The mRNA expression of MRP4 reached peak at the 28th day, and it was 6-fold higher in comparison with that at the -2nd day. Ren et al. (2015) used both young rats (aged 3 months) and old rats (aged 12 months) to illuminate age-related excretion of metformin. The values of t_1/2_, accumulation in urine, and total clearance (CL_tot_) (1.717 ± 0.30 h, 6,088.402 ± 931.55 μg, and 93.414 ± 11.47%, respectively) in young rats were significantly different from those indexes in old rats (2.002 ± 0.51 h, 4,287.087 ± 458.08 μg, and 57.161 ± 18.59%, respectively). A significant age-related decrease of OCT2 is probably responsible for renal excretion of metformin (Ren et al., 2015). Although the lives of experimental animals are much shorter than that of human, we still clarity that the drug transporter undergoes a dynamic change throughout the life. Thus, drug transporters should receive close attentions at fetal, neonatal, mature, and old ages during drug development and clinical use (Andreollo et al., 2012; Sengupta, 2013; Gleeson et al., 2021; Vinarov et al., 2021).

## Effect of Diseases on Transporter Expression and Function

### Effect of Renal-Related Diseases on Drug Transporters in Kidney

Kidney diseases, such as chronic kidney disease (CKD), acute kidney injury (AKI), and renal failure, can change the excretion rate of endogenous and/or exogenous substances, mainly due to a decrease in the glomerular filtration rate and the changes in drug transporters and metabolic enzyme activities. Levels of renal drug transporters are crucial for drug excretion, as drugs were mainly excreted through these transporters. As shown in Table 3, the mRNA and/or protein levels of drug transporters in renal-related disease models were summarized.

**TABLE 3:** The changes of main drug transporters in kidney related diseases on mRNA and/or protein levels.

Pathological state	Specie	Model establishment	OAT1	OAT3	OATP4C1	OCT2	MDR1	BCRP	MATE1	MATE2-K	MRP2	MRP4	Reference
Hyperuricemic nephropathy	mice	Mice were intraperitoneally injected with potassium oxonate (300 mg/kg) once daily for 7 days	protein ↓	protein ↓	—	—	—	protein ↓	—	—	—	—	Zhao et al. (2021)
	male SD rats	Rats were oral administrated of adenine (0.1 g/kg) and potassium oxonate (1.5 g/kg) daily for 3 weeks	mRNA ↓	mRNA ↓	—	—	—	—	—	—	—	—	Wang et al. (2020b)
	male ICR mice	Mice were oral administrated with hypoxanthine (300 mg/kg) and oteracil potassium (300 mg/kg) for 10 days	protein ↓	—	—	—	—	—	—	—	—	—	Li et al. (2020)
	male SD rats	Rats were administrated with yeast pellets and adenine (50 mg/kg) for 5 weeks	mRNA ↓	—	—	mRNA ↓	—	mRNA ↓	—	—	—	—	Wang et al. (2019a)
			protein ↓	—	—	protein ↓		protein ↓	—	—	—	—	—
	male SD rats	Rats were oral treated with adenine (100 mg/kg) and ethambutol (250 mg/kg) by once daily for 3 weeks	protein ↓	—	—	protein ↓	—	protein ↓	—	—	—	—	Wu et al. (2017)
	male SD rats	Rats were orally treated adenine (0.1 g/kg) and potassium oxonate (1.5 g/kg) once daily for 3 weeks	protein ↓	protein ↓	—	—	—	—	—	—	—	—	Liu et al. (2017)
	male SD rats	Rats were orally treated with adenine (0.1 g/kg) and potassium oxonate (1.5 g/kg) daily for 3 weeks	mRNA ↓	mRNA ↓	—	—	—	—	—	—	—	—	Liu et al. (2015)
	male SD rat	Rats were orally treated with tacrolimus (1.5 mg/kg) for 28 days	protein ↓	protein ↔	—	—	—	—	—	—	—	—	Kim et al. (2017)
	male KM mice	Mice were was injected intraperitoneally with potassium oxonate (250 mg/kg) once a day for 7 days	mRNA ↓	mRNA ↓	—	—	—	—	—	—	—	—	Zhang et al. (2020)
Nephrotoxicity	male C57/BL6J mice	Mice were injected intraperitoneally with cisplatin (20 mg/kg) at once	—	—	—	mRNA ↓	—	—	mRNA ↓	—	—	—	Freitas-Lima et al. (2020)
						protein ↓			protein ↓				
	male ICR mice	Mice were given a single dose of cisplatin (20 mg/kg)	protein ↓	protein ↓	—	—	—	—	—	—	—	—	Deng et al. (2020b)
	SD rats	Rats were injected intraperitoneally of cisplatin (8 mg/kg)	—	—	—	protein ↑	—	—	—	—	—	—	Jiang et al. (2019)
	male Wistar rats	Rats were received intraperitoneal injection of cisplatin (12 mg/kg)	mRNA ↓	mRNA ↓	—	—	—	—	—	—	—	—	Wang et al. (2018a)
			protein ↓	protein ↓									
	male SD rats	Rats were given cisplatin (7 mg/kg i.p.) at once	—	—	—	mRNA ↑	—	—	—	—	—	—	Soetikno et al. (2019)
	male C57BL/6 J mice	Mice received a single dose of cisplatin (18 mg/kg)	mRNA ↓	mRNA ↔	—	mRNA ↓	mdr1a mRNA ↑ mdr1b mRNA ↑ protein ↓	mRNA ↔	mRNA ↔	mRNA ↔	mRNA ↑	mRNA ↑	Aleksunes et al. (2008)
						protein ↓		protein ↔			protein ↑	protein ↑	
	male Wistar rats	Rats were oral administrated with AA (10 and 20 mg/kg/d) for 7 days	mRNA↓, protein ↔	mRNA ↓	—	mRNA ↓	—	—	—	—	—	—	Lou et al. (2011)
				protein ↓		protein ↓							
	male C57BL/6J mice	Mice were given 0.28 M NH_4_Cl/2% sucrose for 2 or 7 days	mRNA and protein, the 2nd day ↓, the 7th day ↔	—	—	—	—	—	—	—	—	—	Gottier Nwafor et al. (2020)
	male SD rats	Rats were treated with doxorubicin (15 mg/kg; i.p.) at once	—	—	—	—	—	mRNA↓	—	—	—	—	Karimian Pour and Piquette-Miller (2020)
	male Wistar rats	Rats were intraperitoneally injected methotrexate (7 mg/kg) for 3 days	mRNA ↓	mRNA ↓	—	—	—	—	—	—	—	—	Liu et al. (2018)
			protein ↓	protein ↓									
	kidney slices	methotrexate (10 μM)	mRNA ↓	mRNA ↓	—	—	—	—	—	—	—	—	Liu et al. (2018)
			protein ↓	protein ↓									
	male Wistar rats	Rats were intraperitoneally injected gentamicin (100 mg/kg) for 7 days	protein ↓	protein ↔	—	protein ↓	protein ↔	protein ↑	protein ↓	—	protein ↑	protein ↑	Ma et al. (2018)
	rats	Rats were administered intraperitoneally with endotoxin (5 mg/kg)	—	—	—	mRNA ↓	—	—	mRNA ↓	—	—	—	Karimian Pour and Piquette-Miller (2018)
	male Wistar rats	Rats were injected with HgCl_2_ (4 mg/kg) at once	protein ↑	protein ↑	—	—	—	—	—	—	protein ↑	—	Hazelhoff and Torres (2021)
	male Wistar rats	Rats were treated with a single injection (s.c.) of HgCl_2_ at a dose of 5 mg/kg	protein ↓	protein ↓	—	—	—	—	—	—	—	—	Brandoni and Torres (2015)
	male Wistar strain rats	Rats were injecting rats with CdCl_2_ (2 mg Cd/kg/day) for 14 days	mRNA ↓	mRNA ↓	—	mRNA ↓	—	—	—	—	—	—	Ljubojević et al. (2016)
			protein ↓	protein ↓		protein ↓							
	SD rats	Rats were orally administrated with Zuotai (30 mg/kg), HgS (30 mg/kg), HgCl_2_ (34.6 mg/kg)) and MeHgCl (3.2 mg/kg) for 7 days	mRNA in HgCl_2_ group ↓	mRNA ↔	mRNA in HgCl_2_ group ↓	mRNA ↔	mRNA of MDR1b in HgCl_2_ and MeHgCl group ↑	mRNA ↔	mRNA ↔	mRNA ↔	mRNA in HgCl_2_ and MeHgCl group ↑	mRNA in HgCl_2_ and MeHgCl group ↑	Zhang et al. (2017a)
	KM mice	Mice were treated orally with Zuotai (54% β-HgS, 30 mg/kg), α-HgS (30 mg/kg), HgCl_2_ (33.6 mg/kg), MeHgCl (3.1 mg/kg) for 7 days, respectively	mRNA ↔	mRNA in MeHgCl group ↓	mRNA in MeHgCl and HgCl_2_ group ↓	—	—	—	—	mRNA in HgCl_2_ and MeHgCl group ↑	mRNA in HgCl_2_ and MeHgCl group ↑	mRNA in HgCl_2_ and MeHgCl group ↑	Liu et al. (2016)
Ischemia-Reperfusion in kidney	female SD rats	Rats model was induced by bilateral clamping of renal arteries for 45 min	mRNA 6 and 24 h ↓, 72 h ↔	mRNA 6 and 24 h ↓, 72 h ↔	—	—	—	—	—	—	—	—	Schneider et al. (2007)
	male SD rats	Rat model was induced by vascular clamps over both pedicles for 30 min	mRNA ↓	mRNA ↓	—	—	protein ↔	—	—	—	—	—	Matsuzaki et al. (2007)
			protein ↓	protein ↓									
	male SD rats	Rats were induced using vascular clamps over both pedicles for 30 min	mRNA ↓	mRNA ↓	—	mRNA ↓	—	—	mRNA ↓	—	—	—	Matsuzaki et al. (2008)
			protein ↓	protein ↓		protein ↓			protein ↓				
	male Wistar rats	Rats were induced by occluding renal pedicles for 60 min	protein ↓	protein ↓	—	—	—	—	—	—	—	—	Brandoni and Torres (2015)
	male FVB mice	Mice was induced by bilateral clamping of the renal artery and vein for 30 min	—	—	—	—	protein ↓	mRNA ↓	—	—	protein ↓	protein ↓	Huls et al. (2006)
								protein ↓					
Chronic Renal Failure	Male SD rats	model rats were induced by two-stage 5/6 nephrectomy	mRNA ↓	mRNA ↓	—	—	—	—	—	—	—	—	Kong et al. (2017)
	Wistar rats	a 5/6 nephrectomy	—	—	—	—	—	mRNA ↓	—	—	—	—	Nagura et al. (2016)
								protein ↓					
	Male SD rats	Rats were undergone subtotal nephrectomy operation (80% renal ablation)	—	—	—	—	protein 3 and 6 weeks ↓	—	—	—	protein 3 weeks ↓, 6 weeks ↔	—	Laouari et al. (2001)
	male Wistar albino rats	model rats were undergone 5/6 nephrectomy operation	protein ↔	protein ↔	—	protein ↓	—	—	—	—	—	—	Ji et al. (2002)
Nephrotic Syndrome	male SD rats	rats were intravenously injected with adriamycin (6 mg/kg) for once	mRNA and protein, 6, 9 and 12 weeks ↓	—	—	mRNA and protein, 6 and 9 weeks ↓, 12 weeks ↑	mRNA and protein, 6 weeks ↓, 9 weeks ↔ and 12 weeks ↔	—	—	—	mRNA and protein, 6 and 9 weeks ↓, 12 weeks ↑	mRNA and protein, 6 and 9 weeks ↓, 12 weeks ↑	Dong et al. (2020)
Obstructive nephropathy	male Wistar rats	The ureteral obstruction was released after 24 h	protein ↓	protein ↓	—	—	—	—	—	—	—	—	Brandoni and Torres (2015)
Renal transplantation	male LBN and LEW rats	kidneys of LBN rats were transplanted into LEW rats	—	—	—	mRNA and protein in allogeneic transplantation ↓	—	—	—	—	—	—	Ciarimboli et al. (2013)

SD rats: Sprague-Dawley rats; KM mice: kunming mice; FVB mice: Friend leukaemia virus B strain mice; LBN rats: lewis brown norway rats; LEW rats: lewis rats; ↓: decrease in expression; ↑: increase in expression; ↔: no significant difference; /: not mentioned.

Uric acid nephropathy, mainly caused by hyperuricemia, is a common kidney disease associated with hypertension, proteinuria, oedema, etc. (Perry et al., 1976). In animal experiments, adenine and potassium oxonate are often employed to construct uric acid nephropathy models for drug screening, development, and drug interaction research (Liu et al., 2017; Wu et al., 2017; Wang et al., 2020b). To date, OAT1 and OAT3 are the two most studied drug transporters in uric acid nephropathy, as these proteins are responsible effects for uric acid excretion. Reduced OAT1/3 mRNA and protein levels are consistently noted in uric acid nephropathy (Liu et al., 2015; Liu et al., 2017; Wang et al., 2020b; Zhang et al., 2020). The same trend was observed in the uptake transporter OCT2 and efflux transporter BCRP (Wu et al., 2017; Wang et al., 2019a). Nephrotoxic substances, such as cisplatin, gentamicin, and heavy metals, are causative agents of AKI in clinic. In the cisplatin-induced nephrotoxicity model, the mRNA and protein levels of renal OAT1/3 were decreased in both rats and mice, which could prevent the absorption of cisplatin from blood into renal tubular epithelial cell (Wang et al., 2018a; Deng et al., 2020b). MATE1 is an efflux transporter that mediates the excretion of cisplatin from renal tubules into urine. Previous study has proved that the survival time of Mate1^−/−^ mice was significantly shorter than that of the control group. After cisplatin administration, the blood concentration and accumulation in the kidney of Mate1^−/−^ mice were higher than that in the control mice. Using combination of cisplatin and the MATEs inhibitor pyrimethamine was found to be more nephrotoxic than using cisplatin alone. These results suggest that MATE1 mediates the renal excretion of cisplatin and participates in its nephrotoxicity (Kusuhara et al., 2011; Song et al., 2016). However, OCT2 mRNA and protein levels exhibited the species differences, which were decreased in mice but were increased in rats (Aleksunes et al., 2008; Soetikno et al., 2019). It is worth noting that mRNA and protein levels may also show different trends during the course of disease. For instance, oral administration of NH_4_Cl caused downregulation of OAT1 mRNA and protein levels on the 2nd day, but the levels returned to its original levels on the 7^th^ day (Gottier Nwafor et al., 2020). In the model of heavy metal-induced nephrotoxicity, the uptake transporters OAT1/3, OATP4C1, and OCT2 showed decreasing trends, whereas the efflux transporters MDR1, MATE2-K, and MRP2/4 showed increasing trends (Liu et al., 2016; Ljubojević et al., 2016; Zhang et al., 2017a), which decreased absorption, enhanced urinary clearance of heavy metals, and prevented accumulation in the kidney. Ischemia-reperfusion injury (IRI) in the kidney is another most common clinical cause of AKI, which often occurs during the clinical course of shock, acute renal artery occlusion, or renal transplantation. At present, the nonclinical IRI model is usually constructed by sealing the bilateral renal pedicle with a mini-artery clip (Shiva et al., 2020). Interestingly, the drug transporters OAT1/3, OCT2, MDR1, MATE1, and MRP2/4 in this model showed reducing trends. Schneider et al. (2007) reported that the OAT1/3 levels are associated with recovery time after IRI in kidney. Compared with the sham group, OAT1/3 mRNA and protein levels were completely restored to the preoperative level after 7 days postischemic reperfusion. Given that renal OAT1/3 play significant roles in the excretion of many anionic drugs, IRI induced AKI changes the pharmacokinetics of these drugs, including increases in plasma levels and t_1/2_. An animal model with 5/6 nephrectomy is often used to simulate chronic renal failure in humans. Previous studies have reported the downregulation of uptake transporters OAT1/3 and OCT2, as well as the downregulation of efflux transporters MDR1, BCRP, and MRP2 in animal kidneys during the disease statuses (Laouari et al., 2001; Nagura et al., 2016; Kong et al., 2017). Nephrotic syndrome (NS) is a group of clinical symptoms characterized by increased glomerular basement membrane permeability, high proteinuria, hypoproteinemia, high oedema, and hyperlipidemia (Hull and Goldsmith, 2008). During the 12-week experiment, OAT1 mRNA and protein levels consistently exhibited trends of reduction. OCT2 protein level showed a downward trend within 9 weeks, but increased in the 12th week. The same trends were observed in MRP2 and MRP4 (Dong et al., 2020). The unilateral ureteral obstruction (UUO) animal model is often used to simulate clinical obstructive nephropathy. A study reported reduced OAT1/3 protein levels in the UUO model, indicating reduced renal excretion rates and pharmacokinetic changes of some drugs (Brandoni and Torres, 2015). Downregulation of OCT2 protein level was observed in the kidney transplanted rats. Atenolol, pindolol, and ofloxacin are the substrates of OCT2, and their appropriate doses are difficult to achieve in kidney transplanted patients (Ciarimboli et al., 2013).

In summary, in renal-related diseases, the expressions of OAT1/3 transporters are invariably reduced. The OAT1/3 are responsible for the uptake of endogenous and/or exogenous substances from the blood into renal tubular epithelial cells. The reduction in the renal-related diseases is like a double-edged sword. On the one hand, it prevents some toxic substances (e.g., indole sulfate) from entering renal tissue during the kidney injury, subsequently reduces renal damage, and thus plays a role in protecting the kidney. On the other hand, it also reduced drug excretion, resulting in increased drug concentration in plasma, which leads to toxic side effects. Clinical pharmacokinetics of morinidazole has proved that sulfate conjugate of morinidazole M7 was a substrate of OAT1 and OAT3, while the glucuronide conjugates of M8-1 and M8-2 were substrates of OAT3. After intravenous infusion of 500 mg morinidazole, the area under the curve (AUC) values for M7, M8-1, and M8-2 were 15.1-, 20.4-, and 17.4-fold, respectively, higher in patients with severe renal impairment than those in healthy subjects (Zhong et al., 2014; Kong et al., 2017). The expressions of efflux transporters are different in different renal diseases. It is noteworthy that the increased MRP2/4 in nephrotoxic pathology will transfer the toxic substances from renal tubular epithelial cells into the urine to exert the detoxification effect. However, the increased MRP2/4 also promote the transfer of some therapeutic drugs. Therefore, both changes should be carefully considered in the treatment of the renal-related diseases.

### Effect of Liver and Gallbladder—Related Diseases on Drug Transporters in Kidney

The Inner Canon of Huangdi, an ancient Chinese medical scripture that is the basis for traditional Chinese medicine, states that “the liver and kidney are homologous.” This means that, although the structure and function of the liver and kidney are different, they exhibit a close relationship based on physiological and pathological features. For example, renal injury is often also associated with liver-related diseases, such as hepatorenal syndrome (Raj et al., 2020; Simonetto et al., 2020). In Table 4, we have summarized the characteristics of nonalcoholic fatty liver disease (NAFLD), ischemia reperfusion (IR), and bile duct obstruction pathology on renal drug transporters, providing information for optimizing the drug prescriptions. NAFLD is a global disease (with an approximately 25% of global incidence rate) induced by many factors except alcohol and other specific factors, characterized by excessive deposition of fat in hepatocytes (Younossi et al., 2016). Nonclinical NAFLD models have shown that OAT1 mRNA expression was decreased in mice, but no significant difference was found in rats. Elevated OAT3, MDR1, and MPR2/4 mRNA expression levels were observed in both mouse and rat models, but BCRP protein level was decreased (Canet et al., 2015). Clinical treatments for NAFLD typically include metformin, vitamin E, and statins to exert anti-IR, antioxidant, and lipid-lowering effects. Clarke et al. (2015) revealed that metformin exposure is increased in nonalcoholic steatohepatitis mice model (60% of metformin dose excreted in urine) than that in control group (82% of metformin dose excreted in urine), which might be caused by the decreases of OCT2 and MATE1 in the kidneys of disease mice. In patients, 25% of NASH may lead to an impaired kidney function that highlights a need to adjust drug dosage (Targher et al., 2010). A recent research showed that the novel targeting therapeutic agents mainly target peroxisome proliferator-activated receptors (PPARs), farnesoid X receptor (FXR), and glucagon-like-1 (GLP-1) (Zhou et al., 2020b). As one of the subtypes of PPARs, PPARα is widely expressed in the liver, adipose tissue, heart, skeletal muscle, and kidney. Given that PPARα has a regulatory effect on the drug transporters MATE1 and OCT2 (Freitas-Lima et al., 2020), attention should be given to drug disposition *in vivo* during the clinical use of this novel drug simultaneously with other drugs. IRI of the liver is an inevitable complication during liver resection and liver transplantation. The animal model confirmed that hepatic IRI could increase the kidney MRP2/4 levels (Tanaka et al., 2008). Interestingly, the increases of proteins showed obvious delays, compared to those of mRNAs. The increased renal MRP2 accompanied with decreased hepatic MRP2 may protect against oxidative stress and inflammation after hepatic IRI. Extrahepatic cholestasis is a pathophysiological process caused by the obstruction of bile secretion and excretion. This disease status could induce the expressions of the efflux transporter MRP2/4 in the kidney which will promote the excretion of bile acids and some therapeutic drugs (Slitt et al., 2007). OAT1/3 levels are different in various animal models, which may be related to the way and/or time of modeling (Brandoni et al., 2006; Liu et al., 2012). In general, with liver and bile duct-related diseases, the expressions of MRP2/4 are increased, which accelerates the excretion of endogenous/exogenous toxic substances in the kidney to play a protective role in the body.

**TABLE 4:** The changes of main drug transporters in liver and gallbladder related diseases on mRNA and/or protein levels.

Pathological state	Species	Model establishment	OAT1	OAT3	OCT2	MDR1	BCRP	MRP2	MRP4	Reference
Nonalcoholic steatohepatitis	male C57BL/6Jmice	Mice were fed with a methionine choline-deficient diet for 4 weeks	mRNA ↓	mRNA ↔	mRNA ↓	MDR1a mRNA ↑	mRNA ↔	mRNA ↔	mRNA ↑	Canet et al. (2015)
			protein ↔			protein ↑	protein ↔		protein ↑	
	male C57BL/6J mice	Mice were fed with a high-fat diet for 8 weeks	mRNA ↓	mRNA ↔	mRNA ↑	MDR1a mRNA ↑	mRNA ↔	mRNA ↔	mRNA ↔	Canet et al. (2015)
			protein ↔			protein ↔	protein ↓		protein ↔	
	ob/ob mice	Mice were fed with a methionine choline-deficient diet for 4 weeks	mRNA ↓	mRNA ↑	mRNA ↓	MDR1a mRNA ↑	mRNA ↔	mRNA ↔	mRNA ↑	Canet et al. (2015)
			protein ↔			protein ↑	protein ↔		protein ↑	
	db/db mice	Mice were fed with a methionine choline-deficient diet for 8 weeks	mRNA ↓	mRNA ↑	mRNA ↓	MDR1a mRNA ↑	mRNA ↔	mRNA ↑	mRNA ↑	Canet et al. (2015)
			protein ↔			protein ↑	protein ↔		protein ↑	
	male SD rats	Rats were fed with a methionine choline-deficient diet for 4 weeks	mRNA ↔	mRNA ↑	mRNA ↔	MDR1a mRNA ↑	mRNA ↑	mRNA ↑	mRNA ↑	Canet et al. (2015)
			protein ↔			protein ↑	protein ↔	protein ↑	protein ↔	
	male SD rats	Rats were fed with a high-fat diet for 8 weeks	mRNA ↔	mRNA ↔	mRNA ↔	MDR1a mRNA ↔	mRNA ↔	mRNA ↔	mRNA ↔	Canet et al. (2015)
			protein ↔			protein ↑	protein ↔	protein ↔	protein ↔	
	fa/fa rats	Rats were fed with a high-fat diet for 8 weeks	mRNA ↔	mRNA ↔	mRNA ↔	MDR1a mRNA ↔	mRNA ↔	mRNA ↔	mRNA ↔	Canet et al. (2015)
			protein ↔			protein ↔	protein ↓	protein ↔	protein ↔	
Ischemia-reperfusion-induced in liver	male SD rats	Rats were undergone hepatic ischemia for 60 min	—	—	—	—	—	mRNA 3 h ↑, 6 h ↑, 24 h ↔, 48 h ↔	mRNA 3 h ↔, 6 h ↑, 24 h ↔, 48 h ↔	Tanaka et al. (2008)
								protein 3 h ↔,6 h ↑, 24 h ↑, 48 h ↔	protein 3 h ↔, 6 h ↔, 24 h ↑, 48 h ↔	
Extrahepatic Cholestasis	male C57BL/6 mice	Mice were undergone a bile-duct operation for 1, 3, 7, and 14 days	—	—	—	—	—	mRNA 1 day ↑, 3, 7 and 14 days ↔	mRNA ↑	Slitt et al. (2007)
	male Wistar rats	Rats were undergone a bile-duct operation for 21 h	protein in cortex homogenates ↑	protein in cortex homogenates ↑	—	—	—	—	—	Brandoni et al. (2006)
			protein in basolateral membranes ↑	protein in basolateral membranes ↔						
	Male Wistar rats	Rats were undergone a bile-duct operation for 24, 72 and 120 h	—	—	—	—	—	—	protein 24, 72 and 120 h ↑	Tanaka et al. (2002)
	Male Wistar rats	Rats was injected (i.p.) with alpha-naphthylisothiocyanate (50 mg/kg)	mRNA ↓	mRNA ↓	—	—	—	mRNA ↑	—	Liu et al. (2012)
			protein ↓	protein ↓				protein ↑		

SD rats: Sprague-Dawley rats; ob/ob mice: B6.Cg-Lep,ob./J mice; db/db mice: B6.BKS(D)-Lepr,db./J mice; fa/fa rats: Crl:ZUC-Lepr,fa. fatty rats; ↓: decrease in expression; ↑: increase in expression; ↔: no significant difference; /: not mentioned.

### Effect of Metabolic Disease on Drug Transporters in Kidney

As shown in Table 5, metabolic diseases can also cause changes in drug transporters in the kidney. Hyperuricemia is a chronic metabolic disease caused by purine metabolism disorder, and the condition is primarily caused by excessive production and reduced excretion of uric acid. A cross-sectional study in China reported that, with the improvement of people’s quality of life, the prevalence of hyperuricemia has increased from approximately 26.1% in 2010 to approximately 34.4% in 2019 among men in Wuhan city (Wan et al., 2021). High uric acid level is associated with hypertension, CKD, obesity, metabolic syndrome, etc. Therefore, a combined medication is a common treatment for hyperuricemia. Thus, the levels of renal transporters should attract clinician attentions. In animal models, the OAT1/3, OCT2, and BCRP expressions are decreased under the pathological condition, whereas MDR1 and MRP2/4 expressions are not affected by hyperuricemia (Nishizawa et al., 2019; Le et al., 2020). A growing number of studies have reported that OAT1/3, OCT2, and BCRP are involved in urate efflux from epithelial cells to urine (Taniguchi et al., 2021), and these transporters have been extensively studied in the hyperuricemia. Cephalexin is a substrate of OAT1 and is excreted *via* MATE1. Compared with that in control group, the AUC_inf_ of cephalexin was 2.66-fold and the CL_R_ was 0.36-fold in hyperuricemic rats, which were responsible for variations of drug transporters (Nishizawa et al., 2019). Clinically, it is suggested that, in the treatment of the hyperuricemia, it is necessary to pay special attentions to the dosage of the drugs that are primarily excreted by OAT1/3. Streptozotocin is the most commonly used agent to construct animal models of diabetes, due to its selective destruction of islet β cells in animals (Goyal et al., 2016). In the diabetes model, the decreased OAT1/3 and OCT2 expressions and increased BCRP and MRP2/4 expressions were observed in the kidney (Xu et al., 2015a; Zhang et al., 2017b; Wang et al., 2018b). The excretion of antidiabetic drugs could be affected by these changed transporters. For example, metformin is excreted through OCT2, and sitagliptin is transported by OAT3 (Chu et al., 2007; Koepsell, 2013). Therefore, the clinical uses of these drugs during different disease statuses should take their pharmacokinetic changes into consideration. In general, most renal drug transporters are not affected by a long-term administration of a high-fat diet, but decreased expressions of the uptake protein OAT1/3 was observed in the kidney (Lu et al., 2019), which can lead to an accumulation of endogenous toxic substances that may contribute to obesity-related diseases, such as hyperlipidemia, NAFLD, CKD, and diabetes.

**TABLE 5:** The changes of expression of main drug transporters in metabolic disease on mRNA and/or protein levels.

Pathological state	Species	Model establishment	OAT1	OAT3	OCT2	MDR1	BCRP	MATE1	MATE2-K	MRP2	MRP4	Reference
hyperuricemia	male SD rats	Rats were given drinking water with 10% fructose for 6 weeks	mRNA ↓	mRNA ↓	—	—	mRNA ↓	—	—	—	—	Le et al. (2020)
			protein ↓	protein ↓			protein ↓					
	male SD rats	Rats were orally administered with lipid emulsion (10 ml/kg) once daily for 8 weeks	—	—	—	—	protein ↓	—	—	—	—	Chen et al. (2020)
	male SD rats	Rats were intraperitoneally injected with potassium oxonate (200 mg/kg) at once	mRNA ↔	mRNA ↔	—	—	mRNA ↑, protein ↔	—	—	—	—	Niu et al. (2020)
			protein ↔	protein ↔								
	male KM mice	Mice were continuously administrated with potassium oxonate and adenine for 21 days	protein from the 3rd to 21st day, ↓	—	—	—	protein from the 3rd day to 21st day ↓	—	—	—	—	Wen et al. (2020)
	male Swiss mice	Mice were injected intraperitoneally potassium oxonate (250 mg/kg) once a day for 7 days	mRNA ↓	mRNA ↓	—	—	—	—	—	—	—	Alghamdi et al. (2020)
	adult KM mice	Mice were intraperitoneally injected with uric acid (180 mg/kg) at once	mRNA ↔	mRNA ↔	—	—	mRNA ↔	—	—	—	mRNA ↔	Lin et al. (2019)
			protein ↔	protein ↔			protein ↔				protein ↔	
	male Wistar rats	Rats were oral administrated of adenine (0.1 g/kg) and oxonic acid potassium salt (1.5 g/kg) suspended in 0.5% methylcellulose solution daily for 10 days	mRNA ↓	mRNA ↔	mRNA ↓	mRNA ↔	mRNA ↔	mRNA ↓	—	mRNA ↔	mRNA ↔	Nishizawa et al. (2019)
	male and female KM mice	Mice were intraperitoneally injected of oxonic acid potassium salt (300 mg/kg) for 7 days	mRNA ↓	mRNA ↔	—	—	—	—	—	—	—	Fang et al. (2019a)
	male SD rats	Rats were intragastricly administrated of hypoxanthine (200 mg/kg) followed by intraperitoneal injection of oxonic acid potassium salt (200 mg/kg) 1 h later	mRNA ↓	mRNA ↓	—	—	—	—	—	—	—	Fang et al. (2019a)
	Male KM mice	Mice were intraperitoneally injected with potassium oxonate (250 mg/kg) at once	mRNA ↔	—	—	—	—	—	—	—	—	Zhang et al. (2018)
	male SD rats	Rats were intraperitoneally injected with potassium oxonate (600 mg/kg/d) for 2 weeks	protein ↓	protein ↓	—	—	—	—	—	—	—	Wu et al. (2018)
	Male KM mice	Mice were orally administered oxonate (250 mg/kg) once daily for 7 days	mRNA ↓	—	mRNA ↓	—	—	—	—	—	—	Chen et al. (2017)
			protein ↓		protein ↓							
	male Wistar rats	Rats were administered intragastrically with adenine (200 mg/kg) and ethambutol hydrochloride (250 mg/kg) once daily for 10 days	protein ↓	protein ↓	—	—	—	—	—	—	—	Feng et al. (2017)
	male SD rats	Rats were administrated with lipid emulsion (25% lard, 10% cholesterol, 2% sodium deoxycholate, 1% propylthiouracil, 25% Tween-80, and 20% propylene glycol) daily for 8 weeks	mRNA ↓	—	—	—	—	—	—	—	—	Pang et al. (2017a)
	Male KM mice	Mice were intragastricly treated with potassium oxonate (250 mg/kg) for 7 days	protein ↓	protein ↓	—	—	—	—	—	—	—	Chen et al. (2015)
	Male ICR mice	ICR mice were fed with 30% fructose in drinking water for 6 weeks	protein ↓	—	protein ↓	—	protein ↓	—	—	—	—	Yang et al. (2015)
	Male KM mice	Mice were treated with potassium oxonate (250 mg/kg)	protein ↓	—	protein ↓	—	protein ↓	—	—	—	—	Wang et al. (2016)
	male SD rats	Rats were treated with potassium oxonate at 650 mg/kg, ig	protein ↓	—	—	—	—	—	—	—	—	Su et al. (2018)
	Male Wistar rats	Rats were oral administrated with 5% oxonic acid and 2.5% uric acid for 10 days	protein ↓	protein ↓	protein ↓	—	—	—	—	—	—	Habu et al. (2003)
	SD rats	Rats were orally treated with oteracil potassium (300 mg/kg)	—	—	mRNA ↓	—	—	—	—	—	—	Yan et al. (2014)
diabetes	male SD rats	Rats were fed with high fat diet for 6 weeks, and injected with strepotozotocin (30 mg/kg)	—	—	—	—	mRNA ↑	mRNA ↔	—	mRNA ↑	mRNA ↑	Wang et al. (2018b)
	SD rats	Rats were intraperitoneally injected with streptozotocin (70 mg/kg) at once	—	—	—	—	protein ↑	—	—	—	—	Toyoki et al. (2017)
	male Ins2Akita mice	Ins2Akita mice could develop spontaneously into Type 1 diabetes	mRNA ↓ protein ↔	mRNA ↓ protein ↓	mRNA ↓ protein ↔	—	—	—	—	—	—	Xu et al. (2015a)
	ob/ob mice	Mice were received LabDiet 5K20 food for 3 weeks	—	—	—	—	—	—	—	—	mRNA ↑ protein ↑	Cheng et al. (2008)
	C57BL/6J mice	Mice were treated with a high-fat diet and streptozotocin (100 mg/kg)	mRNA ↓	mRNA ↓	—	—	—	—	—	—	—	Zhang et al. (2017b)
	male SD rat	Rats were treated with 10% w/v fructose solution for drinking *ad libitum*	—	—	protein ↓	—	—	—	—	—	—	Rukavina Mikusic et al. (2018)
Obesity	Male SD rats	Rats were treated with high fat pellet diet for 14 weeks	—	—	mRNA ↔ protein ↓	mRNA ↔ protein ↔	—	mRNA ↔ protein ↔	—	—	—	Abdussalam et al. (2017)
	Female C57BJ/6J mice	Mice were treated to a high-fat diet for 16 weeks	—	—	mRNA ↑	—	—	mRNA ↔	mRNA ↔	—	—	Gai et al. (2016)
	C57BL/6J mice	Mice were received a High-Fat Diet for 4, 12 and 24 weeks	mRNA and protein in 4 and 12 weeks ↓; 24 weeks ↔	mRNA and protein in 4 weeks ↓; 12 weeks ↔; in 24 weeks↓	mRNA and protein in 4, 12 weeks ↑; 24 weeks ↔	Mdr1a 4 weeks ↓; 12 weeks ↔; 24 weeks ↓; 24 weeks ↔	—	—	—	—	mRNA and protein in 4, 12 and 24 weeks ↔	Lu et al. (2019)
	obese patients	Asian patients with minimal change disease of the kidney	—	—	protein ↑	—	—	—	—	—	—	Gai et al. (2016)

SD rats: Sprague-Dawley rats; KM mice: kunming mice; FVB mice: Friend leukaemia virus B strain mice; LBN rats: lewis brown norway rats; LEW rats: lewis rats; ↓: decrease in expression; ↑: increase in expression; ↔: no significant difference; /: not mentioned.

### Effect of Other Diseases on Drug Transporters in Kidney

Rheumatoid arthritis (RA) is an autoimmune disease mainly characterized by synovitis and with a prevalence of 0.24% in the world. This disease could decrease the protein level of OAT1, which may lead to an increased t_1/2_ for some drugs (Wang et al., 2020a). In the high-altitude condition, the efflux drug transporters of MDR1 and MRP2 were increased, and clinicians should take it into consideration when drug prescription (Luo et al., 2017). It is noteworthy that different viral infections bring different changes of the renal transporter levels. The poly I:C infected pregnant rat showed lowered levels of OAT1/3, OATP4C1, BCRP, and MRP2 (Karimian Pour and Piquette-Miller, 2020), but the HIV-1 transgenic rat showed reduced OCT2 and MATE1 levels (Karimian Pour and Piquette-Miller, 2018). A hepatitis B virus infection could also damage kidneys, and it could downregulate some kidney transporters (Zuo et al., 2020). This difference should call attentions to pharmacokinetic performance shift of the used drug when different virus-infection in clinic. In an acute lymphoblastic leukemia mice model, the MRP 2/4 levels and the clearance of MRP-mediated drugs were significantly reduced, but the MDR1, OAT3, and OCT2 levels were increased, corresponding to the increased clearances of MDR1-mediated digoxin, OAT3-mediated furosemide, and OCT2-mediated metformin (Zhou et al., 2020a). A study reported that female obese Zucker spontaneously hypertensive fatty rats showed a strongly decreased expression of OAT2 in the kidneys, and OAT2-mediated furosemide renal excretion could be impaired in these rats (Babelova et al., 2015). There may be other diseases affecting the levels of renal transporters and needing further explorations.

## Molecular Docking Method of Drug-Drug Interactions

Currently, polypharmacy has become a common treatment in clinic. If two or more drugs are secreted simultaneously through the same drug transporters in the renal tubules, adverse drug events may increase due to DDIs. It has been reported that the incidence of DDIs can be as high as 8.34–100% of hospital admissions, which has attracted worldwide attention (de Oliveira et al., 2021). For instance, tenofovir, a representative substrate of OAT1, is a widely used antiviral drug. A clinical investigation showed that, when it was used with *para*-aminosalicylic acid (PAS, a long-standing antibiotic), the values of C_max_ and AUC_0–t_ of tenofovir were increased to approximately 3-fold in the co-administration group than that in the alone group. In contrast, the cumulative amount of tenofovir in urine (Ae) was 20.87 ± 5.60 mg when alone use, and that is 7.45 ± 2.56 mg when it was co-administered with PAS (Parvez et al., 2021). Therefore, fast and accurate prediction of DDIs will effectively reduce unexpected clinical adverse events. Over the past decades, the FDA has issued a series of technical guidelines for DDI research, and the Center for Drug Evaluation of China has listed the common drugs as substrates and inhibitors (Sudsakorn et al., 2020; Center for Drug Evaluation and NMPA, 2021). Of note, in addition to *in vivo* and *in vitro* methods, an *in silico* approach has been officially promoted in the latest guidelines (Sudsakorn et al., 2020). Since it is not feasible to test all drugs for DDIs *in vivo* and/or *in vitro*, *in silico* modeling has become a useful tool to predict DDIs and help prescribing drugs.

**FIGURE 3:**
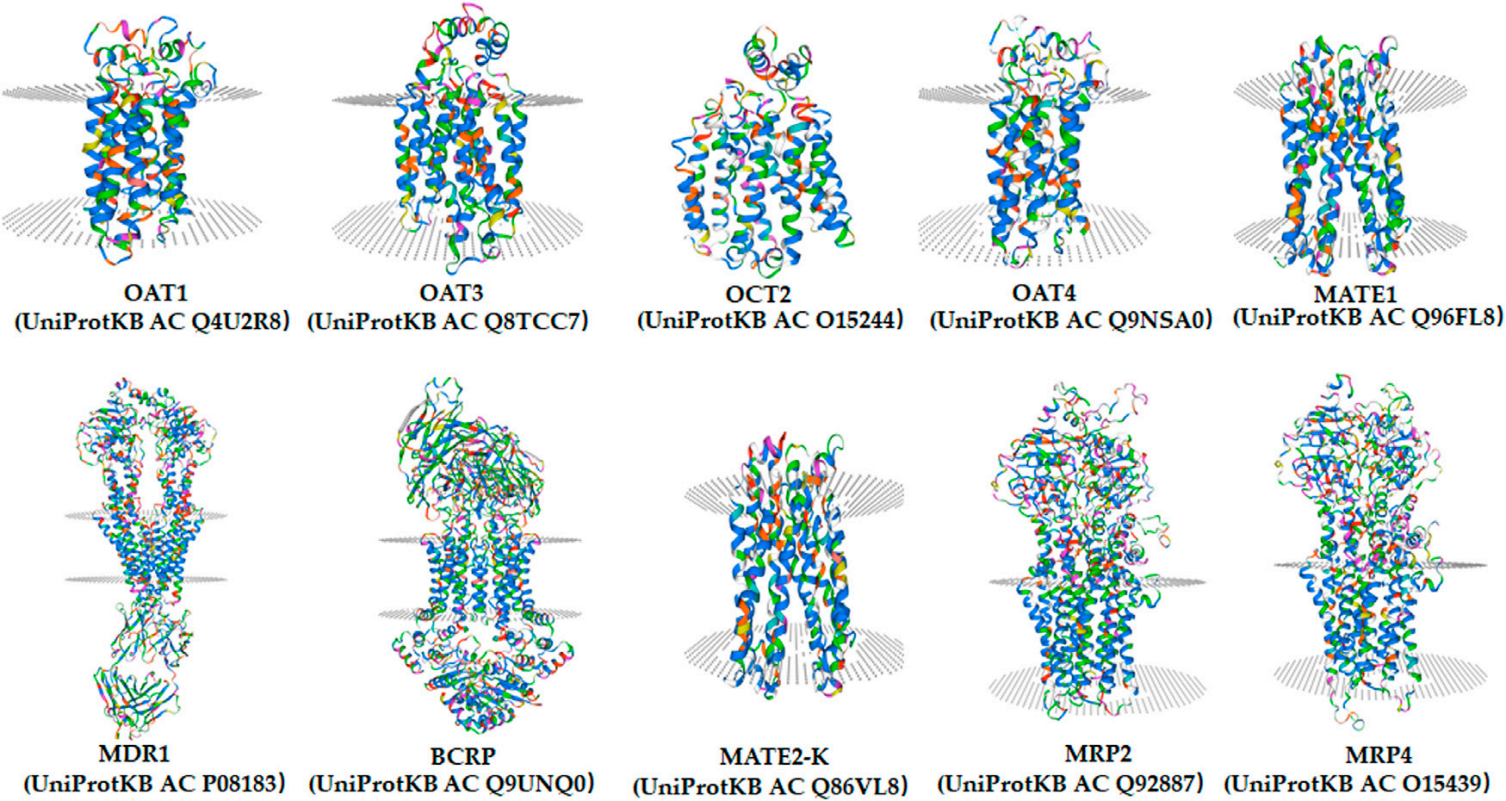
The homology models of OAT1, OAT3, OCT2, MDR1, BCRP, MATE1, MATE2-K, OAT4, MRP2, and MRP4 in human from SWISS-MODEL (https://swissmodel.expasy.org/).

**FIGURE 4:**
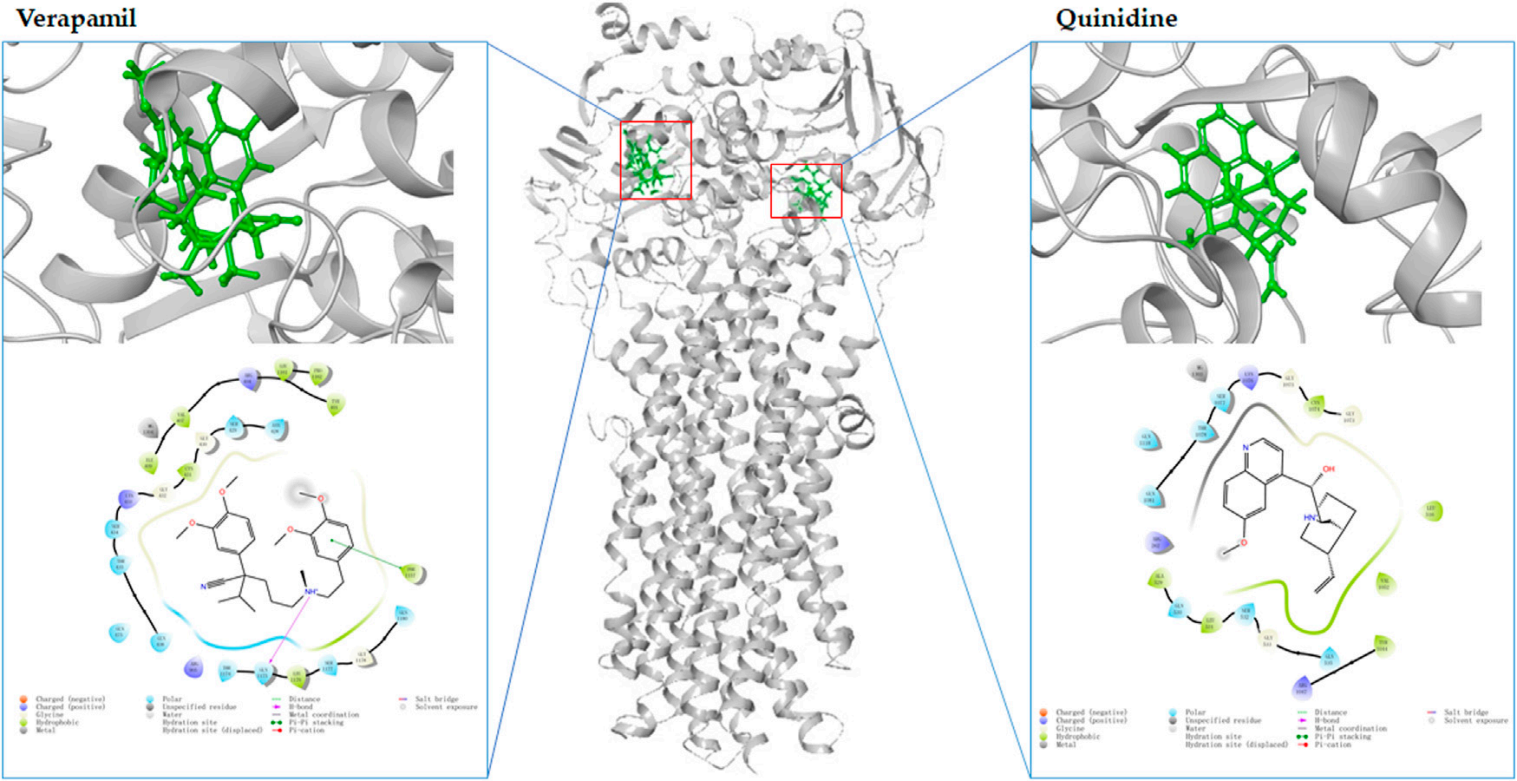
The molecular docking of MDR1 (gray, PDB:6C0V) with verapamil (green left) and quinidine (green, right) by MAESTRO Schrödinger released in 2017-1.

Molecular docking is an *in silico* method based on the “lock-and-key principle” that is used to find the best match between small molecule (ligand) and biological macromolecule (receptor) *via* electrostatic interaction, hydrophobic interaction, van der Waals force interaction, etc. (Pinzi and Rastelli, 2019). It consists of three interrelated parts: binding site recognition, conformational search algorithm, and scoring function. Binding site recognition refers to the identification of the active site in the target protein molecule that interacts with the ligand. Conformational search algorithm is to search the position, orientation, and conformation of ligand with some optimization algorithm only considering that ligand molecules are flexible. The scoring function is the evaluation of the combined conformation in the search (Kitchen et al., 2004). To date, molecular docking has been proven to be extremely useful in identifying substrate and/or inhibitor of drug transporters in a large group of compounds (Ai et al., 2015). A three-dimensional structure is a prerequisite for molecular docking. As shown in Figure 3, the homology models of OAT1, OAT3, OCT2, MDR1, BCRP, MATE1, MATE2-K, OAT4, MRP2, and MRP4 have been established (https://swissmodel.expasy.org/). Taking the MDR1 protein and its substrates and inhibitors as an example (Figure 4), the process of molecular docking is as follows: defining the binding site of the receptor protein, placing the small molecule at the binding site, and then searching for the optimal conformation of the interaction between the small molecule and the macromolecule by continuously optimizing the relative position, conformation, the side chain, and amino acid residues of the receptor. Ultimately, the scoring function is used to identify the ideal binding conformation with the highest score, in which the binding free energy of the receptor and ligand is the lowest and the affinity activity is the highest. Molecular docking provides a rapid method for a priori identification of potential transporter-mediated DDIs and/or drug-induced organ injury.

A large number of clinical events have demonstrated that the modulation of drug transporter activity could easily increase the potential risks of DDIs. Based on this notion, the FDA has explicitly stated that, when a drug under development is a transporter substrate and/or inhibitor, its interaction with a transporter should be evaluated (Sudsakorn et al., 2020). As shown in Table 6, a large number of compounds, especially natural products, have been assessed as drug transporter substrates and/or inhibitors using molecular docking models. For instance, flavonoids are widely found in foods, such as fruits and vegetables. Bai et al. (2019) determined the inhibitory effects of 75 flavonoids on MDR1 protein, and confirmed that at least five flavonoids had significant inhibitory effect on MDR1, which was manifested in the increase of AUC and C_max_ of digoxin when co-administration of digoxin and five flavonoids. Fan et al. (2019) have revealed the inhibitory effects of 99 flavonoids on BCRP, and found that 11 flavonoids had significant inhibitory effect on BCRP, which was manifested in the increase of AUC_0-t_ of mitoxantrone when co-administration of mitoxantrone and these flavonoids. Chemical structure is the determining factor in binding the compound to the target protein, but it must be pointed out that many other factors, such as dose, genetics, and disease status, also play a role in the final biological effect of the compounds. In addition, the molecular docking was simulated independently by computer, and the potential involvement of other proteins from related pathways has not been considered. Therefore, although molecular docking provides a rapid screening method for the study of DDIs, it is essential for *in vitro* and *in vivo* experimental verification.

**TABLE 6:** Summary of published molecular docking models for assessing the substrates and inhibitors of drug transporters.

Transporters	Type of compounds	No. of compounds for test	Descriptors	Reference
OAT1	inhibitors	22 natural compounds	A eukaryotic phosphate transporter from *Piriformospora indica* (Protein Data Bank ID: 4J05) was used as OAT1	Wang et al. (2018c)
	substrates	2 aristolochic acid analogues	The homology modeling of OAT1 was conducted using SWISS MODEL. Docking was performed using LeDock software	Ji et al. (2020)
OAT3	inhibitors	22 natural compounds	The structure of *Escherichia coli* multidrug resistance transporter MdfA (Protein Data Bank ID: 4ZP2) were used as OAT3	Wang et al. (2018c)
	substrates	3 dicaffeoylquinic acids	*Escherichia coli* multidrug resistance transporter MdfA (PDB ID: 4ZP2) was used as OAT3 for homology modeling	Wang et al. (2019b)
OCT2	substrates	14 coupounds	The rbOCT2 sequence was used as a “probe” to search homologous sequences (PSI-BLAST, NCBI database) and sequence-based structural relatives (3D-PSSM)	Zhang et al. (2005)
MDR1	substrates	5 chemotherapeutic drugs	The three-dimensional crystal structure of Mus musculus MDR1 (PDB ID: 4KSB) was used for modeling	Subhani et al. (2015)
	inhibitors	10 acetylated androstano-arylpyrimidines	Homology model of the human MDR1 was obtained from the SWISS-MODEL. Molecular dockings were performed with AutoDock	Gopisetty et al. (2021)
	substrates	3 compounds	The Mdr1p 3D model based on the 3D model of MDR1–6 (Expasy Q9URI1)	Redhu et al. (2016)
	substrates and inhibitors	neochamaejasmin B	The co-crystal of MDR1 (PDB entry: 3G5U) was obtained from the RCSB Protein Data Bank	Eliaa et al. (2020)
	inhibitors	12 oxadiazolothiazin-3-one compounds	The crystal structures of the MDR1 from mouse. three-dimensional model was built	Rosano et al. (2013)
	ligands or inhibitors	21 compounds	P-glycoprotein were created using sequence homologies between Sav186618 and the protein sequence of Pgp (Uniprot entry P08183)	Wise (2012)
	inhibitor	glabridin	The initial structure of P-gp was obtained from the RCSB protein data bank with ID of 4Q9I	Qian et al. (2019)
	substrates and inhibitors	1,1’-([1,1′-Biphenyl]-4,4′-diyl)bis (3-(piperidin-1-yl)propan-1-one)dihydrochloride	Human MDR1 model was corrected using the Prepare Protein tool of DS, and refined with CHARMm	Pang et al. (2017b)
	inhibitors	10 compounds from *Heterotheca inuloides*	The dimensional structures of MDR1 (PDB: 4M1M) was used. The corrected mouse MDR1a (PDB ID: 4M1M) was selected as the template protein	Rodríguez-Chávez et al. (2019)
	substrates	31 drugs	MDR1 (PDB ID: 4Q9H-L) docking model was built with fifty side-chain variants	Mukhametov and Raevsky (2017)
	inhibitors	22 1,2,3,4-Tetrahydroisoquinoline/2H-chromen-2-one conjugates	MDR1 homology model was optimized with AUTODOCK 4.2.6	Rullo et al. (2019)
	inhibitors	21 candidate drugs	The structure of MDR1 was obtained from Protein Data Bank (PDB 6QEX)	Beklen et al. (2020)
	inhibitors	87 natural flavonoids	The three-dimensional structures of all the ligands were prepared in Avogadro. the mouse MDR1 (PDB IDs: 3G60 and 4M1M) and three available cryo-EM structures of the human MDR1 (PDB IDs: 6C0V, 6QEE, and 6QEX) were used	Marques et al. (2021)
	substrates	2 flavonoids from *Pistacia integerrima*	The 3-D structure of mice MDR1 was used from protein data bank with 4Q9L	Rauf et al. (2016)
	inhibitors	50 major herbal constituents	The crystal structure of mouse MDR1 (PDB: 3G60) was selected for molecular analysis	Li et al. (2014)
	substrates and inhibitors	51 chemicals	A human MDR1 homology model was established based on the mouse (Mus musculus) MDR1 protein (PDB ID: 3G5U)	Li et al. (2021)
	inhibitors	75 flavonoids	The crystal structure of mouse MDR1 (PDB: 3G60) was used for docking	Bai et al. (2019)
	inhibitors	15 curcumin derivatives	The X-ray crystal structure of murine MDR1 (PDB ID: 4M1M) and in complex with inhibitors QZ59Se-RRR (PDB ID: 4M2S), QZ59Se-SSS (PDB ID: 4M2T) were prepared for experiment	Sagnou et al. (2020)
	inhibitors	6 cardiotonic steroids	Molecular docking was carried out on the crystal structure of mouse P-glycoprotein (PDB code: 3G60)	Zeino et al. (2015)
	inhibitors	3 natural products and 9 3,4-dihydroisocoumarins	The cryo-EM structure of MDR1 (PDB ID: 6FN1) was prepared for molecular docking	Sachs et al. (2019)
	substrates and inhibitors	11 Polyoxypregnanes	X-ray structure of mouse P-glycoprotein (PDB ID: 4M1M) was used as template structure	To et al. (2017)
	substrates	7 dimeric sesquiterpene thioalkaloids	Mouse P-glycoprotein (PDB ID: 4 M1M) was used for analysis	Guragossian et al. (2021)
	inhibitors	4 sponge-derived sipholane triterpenoids	MDR1 structure was prepared using Biopolymer-Prepare protein structure-module within SYBYL 8.0. QZ59-RRR binding site of MDR1 was analyzed	Abraham et al. (2010)
BCRP	inhibitor	11 flavonoids	The cryo-EM structures of human BCRP (PDB mode: 6FFC) was selected for experiment	Fan et al. (2019)
	inhibitors	10 compounds from *Heterotheca inuloides*	The dimensional structures of BCRP (PDB ID: 5NJ3) was used	Rodríguez-Chávez et al. (2019)
	substrates and inhibitors	51 chemicals	Three-dimensional crystal structure of BCRP (PDB ID: 5NJ3) was used for docking	Li et al. (2021)
	inhibitors	11 natural compounds	The target protein (PDB ID: 6ETI) was performed for molecular docking analysis	Banik et al. (2021)
	inhibitors	99 flavonoids	The high resolutions cryo-EM structures of human BCRP (PDB mode: 6FFC) was selected for study	Fan et al. (2019)
	substrates	5 bisbenzylisoquinoline alkaloids	BCRP model is based on the X-ray structure of mouse P-glycoprotein (Protein Data Bank code 3G5U)	Tian et al. (2013)
	substrates	11 molecules	The experiment was performed using the Glide docking engine and the OPLS2005 force field	Garg et al. (2015)
	inhibitors	68 compounds	The cryo-EM structure of the ABCG2 transporter (PDB ID: 5NJ3) was used for experiment	Antoni et al. (2021)
	inhibitors	13 chromone-based molecules	The two-fold symmetry axis of ABCG2 (PDB ID: 6FFC) was used as a putative multidrug-binding site	Roussel et al. (2019)
	inhibitors	13 compounds	The structure of the human ABCG2-MZ29eFab (PDB ID: 6HIJ) was taken for experiment	Roussel et al. (2020)
	substrates and inhibitors	22 compounds	The human BCRP homology model developed in-house were used as templates for molecular docking	Ferreira et al. (2018)
	inhibitors	14 Homo- and hetero-dimerization of indeno [1,2-b]indoles	The cryo-electron microscopy structure of ABCG2 (PDB ID 5NJ3) was used to docking	Guragossian et al. (2021)
	inhibitors	16 differently 6-substituted 4-anilino-2-phenylpyrimidines16	MZ29 (PDB ID: 6FFC) was from Protein Data Bank (PDB) was used for BCRP docking analysis	Silbermann et al. (2021)
MATE1	inhibitors	881 compounds from the literature	The FASTA sequence of human MATE1 was retrieved from NCBI protein sequence database (Accession: Q96FL8) and develop by four steps	Xu et al. (2015b)
	inhibitor	3 compounds	The three-dimensional structure of hMATE1 was predicted using Modeller, based on the NorM-VC (Protein Data Bank ID: 3MKT) X-ray crystal structure data	Goda et al. (2021)
hMATE2K	inhibitor	3 compounds	The three-dimensional structure of hMATE2K was predicted using Modeller, based on the NorM-VC (Protein Data Bank ID: 3MKT) X-ray crystal structure data	Goda et al. (2021)
MRP2	substrates	11 polyphenolic compounds	The Homology modeling of MRP2 were the structure of Caenorhabditis elegans P-gp (PDB code: 4F4C) and the human MRP1 (PDB code: 2CBZ)	Fang et al. (2019b)
	substrates	44 compounds	MRP2 was modelled on the template of bovine MRP1 bound to leukotriene C4 (PDB ID: 5UJA)	Deng et al. (2020a)
MRP4	substrates and inhibitors	10 endogen substrates, 12 drug substrates, and 16 drug inhibitors	The structure of MRP4 was performed using its primary sequence (code: O15439) from UniProt database	Becerra et al. (2021)
	substrates	4 model substrates	The homology model of MRP4 was built based on the Xray structure of P-glycoprotein (P-gp) from Mus musculus (PDB ID: 3G5U)	Wittgen et al. (2012)
	substrates	two mycophenolic acid-based compounds	A 3D model of the human MRP4 protein in an inward facing conformation was used for analysis	Berthier et al. (2021)
	substrate	ginsenoside compound K	Homology modeling of MRP4 was built by PSI-BLAST, Clustal Omega and SAVES software	Zhou et al. (2019)

## Conclusion

The systemic exposure of drug is influenced by complex systems, not just age, sex-gender, disease, and drug interactions, and renal drug transporter is one of the important factor determining the systemic exposure. The determinant roles of renal drug transporters have been identified in the renal excretion and disease progression. Their expressions in different species have direct impacts on drug disposal, which subsequently has the potential to influence the clinical efficacy of drugs from animals to the clinic in drug development. The expressions of transporters can vary up to 50-fold among species (e.g., BCRP). Interestingly, transporter expression significantly differs in different species and sexes. For instance, different MDR1 expression patterns were noted in rats and mice of different sexes. Therefore, it is very necessary to assess males and females in preclinical studies. Even in the same individual, the expressions of drug transporters could be decreased or increased under different pathological states. As mentioned, renal diseases, liver diseases, and systemic metabolic diseases could alter OAT1, OAT3, OCT2, MDR1, BCRP, MATE1, MATE2-K, MRP2, and MRP4 mRNA and/or protein levels. Therefore, we hypothesize that the expression levels of transporters can be used as an evaluation index for drug treatment effects. Unfortunately, changes in OATP4C1 and OAT4 in disease statuses have not been reported. Changes in transporter expression or the inhibition of transporter function will affect the pharmacokinetics of drugs *in vivo*, and reduce the therapeutic effect of drugs or increase the adverse reactions with the drug accumulation. Especially for drugs with strong toxicity or narrow therapeutic window, their side effects may even threaten the human life. Thus, the clinical dose of the transporter substrate may need to be adjusted when the level of the transporter changes. When the level of the transporter that promotes drug excretion is lowered, consideration should be given to reducing the dose or extending the duration of administration to avoid adverse reactions. Nevertheless, the adjustment of drug dose also depends on metabolic enzyme activity and other factors that affect the pharmacokinetic processes of drugs. With the wide application of drug combination, how to predict and avoid DDIs caused by drug transporters early has become an important challenge in drug administration. Traditional evaluation methods are mainly used *in vivo* animal models and *in vitro* renal cell line models. The *in silico* method provides an effective approach for rapidly screening of drug transporter substrates or inhibitors. In this paper, a large number of natural products were identified as substrates and/or inhibitors of transporters by molecular docking method. However, crystal structures of some drug transporters are still lacking, and the homology simulation method has inevitable disadvantages. Therefore, considerable work must be performed in the future to understand the structure-activity relationship of the interaction between transporters and drugs.
